# ﻿A revised, annotated checklist of Mexican non-biting midges (Diptera, Chironomidae)

**DOI:** 10.3897/zookeys.1191.117223

**Published:** 2024-02-14

**Authors:** Orestes C. Bello-González, Trond Andersen, Norman Mercado-Silva

**Affiliations:** 1 Universidad Autónoma del Estado de Morelos, Av. Universidad 1001, Col. Chamilpa, C.P. 62209, Cuernavaca, Morelos, Mexico; 2 Department of Natural History, University Museum of Bergen, University of Bergen, P.O. Box 7800, NO-5020, Bergen, Norway; 3 Centro de Investigación en Biodiversidad y Conservación (CIByC), Universidad Autónoma del Estado de Morelos, Av. Universidad 1001, Col. Chamilpa, C.P. 62209, Cuernavaca, Morelos, Mexico

**Keywords:** Biodiversity, Nearctic, Neotropical, transition zone

## Abstract

An updated checklist of Mexican non-biting midges (Chironomidae) is presented. A total of 110 species of Chironomidae are known for Mexico: 52 species in 25 genera belong to the subfamily Chironominae, 30 species in 13 genera to Orthocladiinae, 21 species in nine genera to Tanypodinae, five species in two genera to Telmatogetoninae, and two species in one genus to Diamesinae. In addition, 41 genera without identified species are listed. The highest number of species (29) is recorded from the state of Campeche, while 19 species have been found in Veracruz and 15 in Nuevo León. Few or no records exist for states in Central and Northern Mexico, or those on the Pacific coast. The type localities for 34 species are in Mexico; of these, 27 species (25% of the total number of species recorded in the country) are endemic. Twenty-nine species recorded in Mexico have a Neotropical distribution, 15 a Nearctic distribution, and 39 species are distributed in both the Neotropical and Nearctic regions or more widely. It has been suggested that as many as 1000 species might occur in Mexico; so only a little more than 10% of the expected diversity has so far been recorded.

## ﻿Introduction

Mexico is a megadiverse country (Mittermeier et al. 2011; Mendoza-Ponce et al. 2020). Located in the Nearctic-Neotropical transition area, the north to south orientation of numerous warm, low altitude corridors, and the abundance of mountain chains with colder conditions have allowed biota to disperse during past climate change events (Halffter 1987). This high biodiversity results primarily from an accumulation of taxa from other areas and constant changes in the landscape (Priego Santander and Esteve Selma 2017) rather than local diversification (Sundaram et al. 2019; Harvey et al. 2020). However, several of the most species rich and ecologically relevant insect groups are not included in these studies.

Chironomidae have the widest distribution of all free-living groups of holometabolous insects and are likely the most taxonomically and ecologically diverse family of aquatic insects (Cranston 1995; Dijkstra et al. 2014). Reiss (1982) estimated that between 1500–2000 chironomid species might occur in tropical Mexico and Central America, and Andersen et al. (2000) suggested that as many as 1000 species can be expected to occur in Mexico. Spies et al. (2009) compiled an annotated list of the Mexican and Central American genera including a key to the genera known from the region at that time. However, the latest inventory of the Mexican chironomids only included 61 species plus an additional 25 genera without identified species (Andersen et al. 2000).

During the last two decades, several new species have been described based on material from Mexico (Kyerematen et al. 2000; Kyerematen and Andersen 2002; Wang et al. 2006; Vinogradova 2008; Andersen et al. 2010, 2016; Wiedenbrug et al. 2012; Pinho et al. 2013; Acosta et al. 2017; Pinho and Andersen 2021; Andersen 2023). Several species and genera have also been recorded for the first time from Mexico, especially in connection with surveys of particular habitats like the aquatic fauna in spring-fed tropical canyons in the southern Sonora desert (Bogan et al. 2014), or subfossil Chironomidae in surface sediments of the sinkholes of the Yucatan Peninsula (Hamerlík et al. 2018). Several of these studies are based mainly on larvae and the materials are generally not identified beyond genus level.

The Nearctic-Neotropic transition should lead to the existence of chironomid species with different biogeographic affinities. The Nearctic fauna is comparatively well known (Oliver et al. 1990; Oliver and Dillon 1994) and most chironomids in Mexico with this biogeographic affinity can be identified to genus level using the keys to the larvae, pupa and adults of the Holarctic Region (Wiederholm 1986, 1989; Andersen et al. 2013a). The Neotropical chironomids from Mexico are much less studied and more difficult to identify based on available literature.

An updated checklist of Mexican Chironomidae species is presented. The list provides an updated baseline and will facilitate the study of the chironomid fauna in the Nearctic-Neotropical biogeographical transition zone in Mexico. The checklist is based on Andersen et al. (2000), and new records and species published during the last two decades are added. Some ecological information now available for the genera recorded from Mexico are also included.

## ﻿Methods

The checklist is based on Andersen et al. (2000); references already given in that list are not repeated here. The checklist includes published records only. Records were compiled from peer reviewed scientific articles, books, and book chapters and, to a lesser extent, unpublished project reports. Specimens of Mexican chironomids are housed in several collections (Contreras-Ramos 2021; Huerta Jiménez 2021; Admin 2022; Bentley and Thomas 2022; European Bioinformatics Institute 2022); and these records can be accessed using “Name search” in GBIF (2023).

Following Ashe and O´Connor (2009) eight major zoogeographical regions are recognized: Antarctic (AN), Neotropical (NT), Nearctic (NE), Palaearctic (PA), Afrotropical (AF), Oriental (OR), Australasian (AU), and Oceanian (OC). Administratively, Mexico is divided in 32 states. Of these the 18 northernmost states are generally regarded as belonging to the Nearctic region, while the remaining 13 southern most states as belonging to the Neotropical region (Ashe and O´Connor 2009). However, the biogeographical zones are not clearly defined and depend to some degree on the group of organisms studied. There are also clearly transition zones between the two regions. Given this, taxa present in Mexico and in Central or South America are considered to be Neotropical, while taxa present in Mexico and in the USA and/or Canada are considered to be Nearctic. The exception is taxa from southern Florida, USA, which is considered to be Neotropical. However, many species are found both in South- and North America or have a wider distribution.

The checklist is arranged alphabetically. Species group names follow the genus and subfamily names. A short outline with information on the number of species, distribution, and larvae habitats is given for each genus. For literature records given as “Cricotopuscf.sylvestris” or “*Cricotopussylvestris* group” we assume they are correctly identified to genus level. Following Ashe and O’Connor (2009), two Tanypodinae species originally described as *Macropelopiaroblesi* Vargas, 1946 and *Pentaneuramarmorata* Johannsen, 1938 are listed as “Generically unplaced valid Macropelopiini” and “Generically unplaced valid Tanypodinae”, respectively.

The valid species name is followed by the original combination in parenthesis, with type country (for USA, country and state) in square brackets. When the type locality is situated in Mexico, more specific information is given for the type locality. Synonyms are given if descriptions are based on Mexican material. Mexican records are then given followed by the state(s) from where the species is recorded in square brackets. Finally, the total distribution for a species is given as zoogeographical region(s), followed by the countries from where the species has been recorded. However, this list of countries might not be complete, and for widespread species it is only given as “widespread”.

### ﻿Citations for species names are arranged as follows

***Cladopelmaforcipis* (Rempel, 1939: 211)** (Chironomus (Cryptochironomus)) [Brazil]. Andersen et al. (2000: 590) [Mexico State; Morelos; Veracruz]; Vinogradova and Riss (2007: 34) [“Yucatan Peninsula”]; European Bioinformatics Institute (2022) [Quintana Roo]. NT, NE. Brazil, Colombia, Costa Rica, Guatemala, Mexico, Nicaragua, Panama, USA.

In the checklist, recorded genera lacking identified species are included. Mexican records are listed as e.g.: *Cryptochironomus* sp., followed by the state(s) from where the genus is recorded in square brackets.

Vinogradova and Riss (2007) provided numerous records from the Yucatan Peninsula, but without giving any details on the localities. As Yucatan Peninsula includes partly or totally the territory of three Mexican states; these records are listed as [“Yucatan Peninsula”].

## ﻿Results

### ﻿Check list

**Subfamily Chironominae**

**Genus *Apedilum* Townes, 1945**

A genus with three named species. *A.elachistus* Townes, 1945 is widespread throughout North and South America, *A.subcinctum* Townes, 1945 is distributed in North and Central America, and *A.griseistriatum* (Edwards, 1931) occurs in South America. Larvae are associated with submerged vegetation in ponds, canals, lakes, and slowly running rivers, both in fresh or brackish water (Epler et al. 2013).

***Apedilumelachistus* Townes, 1945: 33** [USA: Texas]. Andersen et al. (2000: 590) [States of Campeche; Puebla; Veracruz]; Contreras-Ramos et al. (2000: 25); Vinogradova and Riss (2007: 33) [“Yucatan Peninsula”]. NT, NE. Argentina (Donato et al. 2008a), Brazil, Canada (Giberson et al. 2001), Costa Rica, Guatemala, Mexico, Nicaragua, Uruguay (Donato et al. 2008a), USA.

***Apedilumsubcinctum* Townes, 1945: 33** [USA: Nevada]. Andersen et al. (2000: 590) [States of Campeche; Jalisco]; Contreras-Ramos et al. (2000: 25); Vinogradova and Riss (2007: 33) [“Yucatan Peninsula”]; Contreras-Ramos (2021). NT, NE. Guatemala, Mexico, USA.

**Genus *Asheum* Sublette & Sublette, 1983**

See: *Polypedilum* Kieffer, 1912.

**Genus *Axarus* Roback, 1980**

A genus of ~ 15 species that occur in the Neotropical, Nearctic, Palaearctic, and the Australasian regions. Ten species are known from South America (Andersen and Mendes 2002a; Andersen et al. 2018; Pinho et al. 2019). Larvae occur in littoral to sublittoral soft sediments in lakes and rivers (Epler et al. 2013).

***Axarusrogersi* (Beck & Beck, 1958: 27)** (*Xenochironomus*) [USA: Florida]. Andersen et al. (2000: 590) [Campeche State]; Contreras-Ramos et al. (2000: 26); Contreras-Ramos (2021). NT, NE. Costa Rica, Mexico, Nicaragua, USA.

**Genus *Beardius* Reiss & Sublette, 1985**

A genus with > 30 named species that occur mainly in tropical areas in the Neotropical region with a few species in the southern parts of the Nearctic region (Jacobsen and Perry 2000; Pinho et al. 2013). The larvae have been found associated with macrophytes or submerged wood in both standing and flowing waters (Epler et al. 2013).

***Beardiusaciculatus* Andersen & Sæther, 1996: 40** [Costa Rica]. Andersen et al. (2000: 590) [States of Campeche; Veracruz]; Contreras-Ramos et al. (2000: 26); Vinogradova and Riss (2007: 33) [“Yucatan Peninsula”]; Contreras-Ramos (2021); Admin (2022). NT. Costa Rica, Mexico.

***Beardiuschapala* Pinho, Mendes & Andersen, 2013: 28** [Mexico: Jalisco State, Lake Chapala, El Chante]. Endemic.

***Beardiusparcus* Reiss & Sublette, 1985: 183** [Venezuela]. Andersen et al. (2000: 590) [Veracruz State]; Contreras-Ramos et al. (2000: 26) [Campeche State]; Contreras-Ramos (2021). NT. Costa Rica, Mexico, Nicaragua, Venezuela.

**Genus *Caladomyia* Säwedal, 1981**

See: *Tanytarsus* Wulp, 1874.

**Genus *Chironomus* Meigen, 1803**

One of the most species-rich and common chironomid genera, with ~ 300 described species from all zoogeographical regions except Antarctica. The larvae graze on detritus or are filter-feeders, predominantly in soft sediments of standing water, rarely in flowing water (Epler et al. 2013).

***Chironomusalchichica* Acosta & Prat in Acosta et al. 2017: 53** [Mexico: Puebla State, Lake Alchichica]. Endemic.

***Chironomusstigmaterus* Say, 1823: 15** [USA: Pennsylvania]. Andersen et al. (2000: 590) [States of Durango; Puebla]; Alcocer et al. (2016: 411). NT, NE. Brazil, Cuba, Mexico, USA.

**Genus *Cladopelma* Kieffer, 1921**

A genus of ~ 20 described species that occur in all zoogeographical regions except Antarctica and Oceania. The larvae live in streams and larger rivers, lakes, and ponds as well as brackish water and hot springs (Epler et al. 2013).

***Cladopelmaforcipis* (Rempel, 1939: 211)** (Chironomus (Cryptochironomus)) [Brazil]. Andersen et al. (2000: 590) [States of Mexico; Morelos; Veracruz]; Vinogradova and Riss (2007: 34) [“Yucatan Peninsula”]; European Bioinformatics Institute (2022) [Quintana Roo State]. NT, NE. Brazil, Colombia, Costa Rica, Guatemala, Mexico, Nicaragua, Panama, USA.

**Genus *Cladotanytarsus* Kieffer, 1921**

A genus of ~ 80 described species that occur in all zoogeographical regions except Antarctica and Oceania. No named species are recorded from South America, but larval morphotypes have been recorded from Brazil (Roque et al. 2004). Larvae construct sessile cases of fine detritus and have been found in streams and larger rivers, lakes, and ponds, as well as in brackish water and hot springs (Epler et al. 2013).

***Cladotanytarsusviridiventris* (Malloch, 1915: 491)** (*Tanytarsus*) [USA: Michigan]. Andersen et al. (2000: 590) [Puebla State]. NE. Canada, Mexico, USA.

**Genus *Cryptochironomus* Kieffer, 1918**

A genus of ~ 60 named species that occur in all zoogeographical regions, except Antarctica. Four species are described from South America (da Silva et al. 2010). Larvae occur on various substrates in lakes, small streams, and larger rivers (Epler et al. 2013).

***Cryptochironomus* sp.**: Vinogradova and Riss (2007: 34) [“Yucatan Peninsula”]; Granados-Ramírez et al. (2017: 45) [Morelos State].

**Genus *Dicrotendipes* Kieffer, 1913**

A genus of ~ 85 described species that occur in all zoogeographic regions except Antarctica. The genus was revised by Epler (1988). The larvae inhabit the littoral sediments of standing waters and may be common in lentic habitats (Epler et al. 2013).

***Dicrotendipesaethiops* (Townes, 1945: 107)** (Tendipes (Limnochironomus)) [USA: New Mexico].

Syn.: Tendipes (Limnochironomus) figueroai Vargas, 1952: 48 [Mexico: Morelos State].

Andersen et al. (2000: 590) [States of Baja California; Mexico; Querétaro]; Huerta-Jiménez (2021) [Morelos State]. NE. Mexico, USA.

***Dicrotendipescalifornicus* (Johannsen, 1905: 217)** (*Chironomus*) [USA: California]. Andersen et al. (2000: 590) [States of Mexico; Morelos; Oaxaca; Sinaloa]; Bentley and Thomas (2022) [Michoacán State]. NT, NE. Chile, Colombia, Costa Rica, Guatemala, Mexico, Panama, Peru, USA.

**Remark.**Bentley and Thomas (2022) recorded the species from “Cojumatlán, Jalisco”. The town of Cojumatlán is located on the shoreline of Lake Chapala in the State of Michoacán. Although close to the border between the two states, the original reference to the State of Jalisco most probably is a mistake.

***Dicrotendipesneomodestus* (Malloch, 1915: 475)** (*Chironomus*) [USA: Illinois]. Andersen et al. (2000: 590) [Puebla State]; Alcocer et al. (2016: 412). NE. Canada, Mexico, USA.

***Dicrotendipesobrienorum* Epler, 1987: 148** [Mexico: Michoacán State, Patzcuaro]. Andersen et al. (2000: 590). Endemic.

***Dicrotendipessinoposus* Epler, 1987: 152** [Mexico: Hidalgo State, Otongo]. Andersen et al. (2000: 590) [States of Campeche; Hidalgo; Veracruz]; Contreras-Ramos et al. (2000: 26); Vinogradova and Riss (2007: 34) [“Yucatan Peninsula”]; Contreras-Ramos (2021). NT. Brazil, Colombia, Costa Rica, Dominica, Mexico, Nicaragua.

**Genus *Einfeldia* Kieffer, 1922**

The concept, content and status of *Einfeldia* have been, and to an extent remain, confusing (Cranston et al. 2016a). Narrowly defined, *Einfeldia* contains approximately five species and is distributed in the Neotropical, Nearctic, Palaearctic, Oriental, and Australasian regions. The larvae inhabit standing, predominantly dystrophic waters (Epler et al. 2013).

***Einfeldia* sp.**: Navarrete-Salgado et al. (2004: 157) [México State]; Vinogradova and Riss (2007: 34) [“Yucatan Peninsula”].

**Genus *Endochironomus* Kieffer, 1918**

A genus of ~ 20 named species distributed in the Nearctic, Palaearctic, Afrotropical, and Oriental regions. The larvae occur in “Aufwuchs” on living and dead substrata in almost all types of still water; they mine leaves and stems of macrophytes preferentially in small, eutrophic standing waters (Epler et al. 2013).

***Endochironomussubtendens* (Townes, 1945: 65)** (Tanytarsus (Endochironomus)) [USA: New York]. Andersen et al. (2000: 590) [Yucatán State]. NE. Canada, Mexico, USA.

**Genus *Endotribelos* Grodhaus, 1987**

A genus of 14 described species, all occurring in the Neotropical and Nearctic regions except one species from China. The Brazilian species were treated by Trivinho-Strixino and Pepinelli (2015). The larvae are associated with aquatic macrophytes, decaying leaves, wood, and fallen fruits in streams (Epler et al. 2013).

***Endotribeloshesperium* (Sublette, 1960: 217)** (Tendipes (Tribelos)) [USA: California]. Andersen et al. (2000: 590) [Puebla State]. NE. Mexico, USA.

**Genus *Fissimentum* Cranston & Nolte, 1996**

A genus with four named species endemic to South America; but larval morphotypes have also been recorded from the Nearctic, Oriental, and Australasian regions (Epler et al. 2013). Larvae are found in fine sediments of lentic and lotic habitats and can tolerate desiccation (Cranston and Nolte 1996).

***Fissimentum* sp.**: Vinogradova and Riss (2007: 33) [“Yucatan Peninsula”].

**Genus *Glyptotendipes* Kieffer, 1913**

The taxonomy and nomenclature of the genus have been confusing since its establishment. *Glyptotendipes* now includes ~ 27 species, distributed in the Neartic, Paleartic, Oriental, and Afrotropical regions (Epler et al. 2013; Konar and Majumdar 2021). Three subgenera are recognized, *Glyptotendipes* s. str. (including *Phytotendipes* Goetghebuer, 1937), *Caulochironomus* Heyn, 1992, and *Heynotendipes* Spies & Sæther, 2004 (including *Trichotendipes* Heyn, 1992) (see Spies and Sæther 2004). Larvae occur in detritus-rich littoral sediments of lakes, ponds, small water bodies, and running water (Epler et al. 2013).

***Glyptotendipes* sp.**: Contreras-Ramos and Andersen (1999: 4) [Campeche State]; Vinogradova and Riss (2007: 34) [“Yucatan Peninsula”].

**Genus *Goeldichironomus* Fittkau, 1965**

A genus of 15 named species mainly distributed in the Neotropical region (tropical and subtropical Central and South America), but several species reach their northern limits in southeastern USA (Donato and Andersen 2022). The larvae of *Goeldichironomus* are mostly found in sediments, on plants or in floating mats of vegetation in lentic habitats, in fresh to brackish water, and in oligotrophic to hypereutrophic conditions (Epler et al. 2013).

***Goeldichironomusamazonicus* (Fittkau, 1968: 260)** (*Siolimyia*) [Brazil]. Andersen et al. (2000: 590) [Veracruz]; Vinogradova and Riss (2007: 34) [“Yucatan Peninsula”]. NT. Bahamas, Brazil, Mexico, Nicaragua, Panama, Peru, USA: Florida, Venezuela, Virgin Islands.

***Goeldichironomuscarus* (Townes, 1945: 118)** (*Tendipes*) [Venezuela]. Vinogradova and Riss (2007: 32) [“Yucatan Peninsula”]. NT, NE. Colombia, Costa Rica, Mexico, Nicaragua, Panama, Venezuela, USA.

***Goeldichironomusholoprasinus* (Goeldi, 1905: 135)** (*Chironomus*) [Brazil]. Andersen et al. (2000: 590) [Tabasco]; Vinogradova and Riss (2007: 34) [“Yucatan Peninsula”]. NT, NE. Argentina, Brazil, Costa Rica, Ecuador, Mexico, Nicaragua, Panama, Peru, USA, Venezuela, Virgin Islands.

**Genus *Harnischia* Kieffer, 1921**

A genus of ~ 20 described species that occur in all zoogeographic regions except the Antarctic, Neotropical, and Oceanian regions. However, unnamed larvae have been recorded from Brazil (Roque et al. 2004). Larvae occur in soft sediments of generally clean lakes and larger rivers (Epler et al. 2013).

***Harnischia* sp.**: Contreras-Ramos et al. (2000: 26) [Campeche State].

**Genus *Hyporhygma* Reiss, 1982**

A genus with a single named species, *H.quadripunctatum* (Malloch, 1915), distributed in eastern North America, from Newfoundland to Florida. The larvae mine leaves and stems of *Nuphar* and *Nymphaea* species (Epler et al. 2013).

***Hyporhygma* sp.**: Vinogradova and Riss (2007: 34) [“Yucatan Peninsula”].

**Genus *Kiefferulus* Goetghebuer, 1922**

Syn.: *Nilodorum* Kieffer, 1921 (see Cranston et al. 1990).

A genus with at least five species in the Holarctic region. Species previously considered to belong to *Nilodorum* are widespread in the Afrotropical, Oriental, and Australasian regions. The larvae inhabit sediments of small to medium sized waterbodies (Epler et al. 2013).

***Kiefferulus* sp.**: Contreras-Ramos et al. (2000: 26) [Campeche State].

**Genus *Lauterborniella* Thienemann & Bause, 1913**

A genus with a single named species, *L.agrayloides* (Kieffer, 1911) distributed in the Neotropical, Nearctic, and Palaearctic regions. Other species referred to as *Lauterborniella* in the literature belong either to *Zavreliella* Kieffer, 1920 or to *Kribiodorum* Kieffer, 1921, or their generic affinities are unclear (Epler et al. 2013). Larvae are mobile amongst submerged vegetation in small bodies of standing water (Epler et al. 2013). In Brazilian streams they have also been found in accumulations of litter attached to stones (Sanseverino and Nessimian 2001).

***Lauterborniella* sp.**: Bogan et al. (2014: 2726) [Sonora State].

**Genus *Microchironomus* Kieffer, 1918**

A genus of approximately ten species distributed in the Nearctic, Palaearctic, Afrotropical, and Oriental regions (Yan and Wang 2006). The larvae occur in lakes, rivers, and ditches, including brackish water (Epler et al. 2013).

***Microchironomusnigrovittatus* (Malloch, 1915: 456)** (*Chironomus*) [USA: Illinois]. Andersen et al. (2000: 590) [Veracruz State]. NE. Mexico, USA.

**Genus *Micropsectra* Kieffer, 1909**

Based on morphological and molecular data, *Krenopsectra* Reiss, 1969 and *Parapsectra* Reiss, 1969 were recently considered to be junior synonyms of *Micropsectra* (Ekrem et al. 2010). The three genera have a Holarctic distribution with ~ 100 valid species. The larvae have been recorded from a wide range of habitats, including hygropetric situations, thermal springs, moorland pools, and temporary pools. They are often found in muddy deposits in slow flowing stretches of streams and small rivers and in mesotrophic and oligotrophic lakes (Epler et al. 2013).

***Micropsectra* sp.**: Vinogradova and Riss (2007: 34) [“Yucatan Peninsula”]; Alcocer et al. (2016: 411) [Puebla State].

**Remarks.** Both records from Mexico appear to be based on larvae only. According to Epler et al. (2013) the larvae of *Micropsectra* can be difficult to separate from *Tanytarsus* larvae.

**Genus *Microtendipes* Kieffer, 1915**

A genus of ~ 55 named species that occur in all zoogeographic regions, except Antarctica; Neotropical records are based only on larvae not identified to species level (Roque et al. 2004). Larvae are found in littoral and sublittoral sediments of lakes, and in sediments and submerged mosses in running water (Epler et al. 2013).

***Microtendipes* sp.**: Vinogradova and Riss (2007: 34) [“Yucatan Peninsula”]; Granados-Ramírez et al. (2017: 45) [Mexico State].

**Genus *Nandeva* Wiedenbrug, Reiss & Fittkau, 1998**

A genus with seven described species that occur in the Neotropical and Australasian regions (Andersen et al. 2011b). The only described larvae was found in semi-immersed leaf litter packs in a tropical stream in Australia (Cranston 2019).

***Nandevastrixinorum* Sæther & Roque, 2004: 67** [Brazil]. Andersen et al. (2011b: 55) [Campeche State]. NT. Brazil, Mexico.

**Genus *Nilothauma* Kieffer, 1921**

A genus with > 60 described species distributed throughout most zoogeographic regions except Antarctica. The Neotropical species were reviewed by Pinho and Andersen (2021). The larvae inhabit littoral and sublittoral soft sediments of lakes, streams, and rivers (Epler et al. 2013).

***Nilothaumamaya* Pinho & Andersen, 2021: 103** [Mexico: Campeche State, Calakmul]. Endemic to Mexico.

**Genus *Nimbocera* Reiss, 1972**

See: *Tanytarsus* Wulp, 1874.

**Genus *Omisus* Townes, 1945**

See: *Zavreliellalongiseta* Reiss, 1990.

**Genus *Oukuriella* Epler, 1986**

A genus of > 20 species restricted to the Neotropical region. The larvae can be found associated with freshwater sponges or submerged wood in streams and rivers (Fusari et al. 2014). Species associated with sponges were revised by Fusari et al. (2014).

***Oukuriellaannamae* Epler, 1996: 4** [Costa Rica]. Andersen et al. (2000: 590) [Campeche State]; Contreras-Ramos et al. (2000: 26). NT. Brazil (Bellodi et al. 2016), Costa Rica, Mexico.

***Oukuriellaoliveirai* Messias & Fittkau, 1997: 256** [Brazil]. Bellodi et al. (2016: 191) [Campeche State]. NT. Brazil, Mexico.

***Oukuriellasimulatrix* Epler, 1986: 160** [Colombia]. Andersen et al. (2000: 590) [Campeche State]; Contreras-Ramos et al. (2000: 26); Vinogradova and Riss (2007: 33) [“Yucatan Peninsula”]; Bellodi et al. (2016: 193). NT. Colombia, Mexico.

**Genus *Parachironomus* Lenz, 1921**

The genus has a worldwide distribution with at least 30 species in the Holarctic region and 20 species in the Neotropical region (Trivinho-Strixino et al. 2010; Epler et al. 2013). The adults of the Neotropical species were revised by Spies et al. (1994). Larvae are found in lentic and lotic water bodies under a wide range of conditions, including leaf miners in submerged macrophytes; they also live in association with Bryozoa or are ectoparasites on other invertebrates (Epler et al. 2013).

***Parachironomusdirectus* (Dendy & Sublette, 1959: 514)** (Tendipes (Cryptochironomus)) [USA: Alabama]. Andersen et al. (2000: 590) [Morelos State]; Vinogradova and Riss (2007: 33) [“Yucatan Peninsula”]. NT, NE. Mexico, Nicaragua, Panama, USA.

***Parachironomushazelriggi* Spies, 2000: 133** [USA: California]. Andersen et al. (2000: 590) as *P.monochromus* Wulp, 1874 [States of Querétaro; Mexico]; Spies (2000: 134) [Guanajuato State; Mexico City]. NE, PA. Canada, Mexico, Russia (Orel 2017: 535), USA.

**Remarks.** According to Spies (2000: 129) *P.monochromus* Wulp, 1874 is today considered to be a Palaearctic species and listing from Mexico following Spies & Reiss (1996: 71) must be changed to *P.hazelriggi*.

***Parachironomustenuicaudatus* (Malloch, 1915: 475)** (*Chironomus*) [USA: Illinois]. Andersen et al. (2000: 590) [Puebla State]. NE, PA. Widespread.

**Remarks.** According to Spies (2000: 133) the record from Puebla is based on Alcocer et al. (1993) and must be considered as uncertain as it appears to be based on immature specimens only.

**Genus *Paracladopelma* Harnisch, 1923**

The genus has a predominantly Holarctic distribution, with at least 20 known species; many species are also recorded from the Oriental region (Epler et al. 2013; Yan et al. 2008). The Holartic species were reviewed by Jackson (1977). Larvae inhabit sandy substrata in lakes, streams, and small rivers and the soft profundal sediments of deep lakes (Epler et al. 2013).

***Paracladopelma* sp.**: Vinogradova and Riss (2007: 33) [“Yucatan Peninsula”].

**Genus *Paralauterborniella* Lenz, 1941**

A genus with two described species; one of them, *P.nigrohalteralis* (Malloch, 1915), is widely distributed (Tang 2016). The larvae usually occur in littoral soft sediments of lakes (Epler et al. 2013).

***Paralauterborniella* sp.**: Vinogradova and Riss (2007: 33) [“Yucatan Peninsula”].

**Genus *Paratanytarsus* Thienemann & Bause, 1913**

A genus of > 60 named species that occur in all zoogeographical regions except Antarctica. The larvae inhabit brackish ponds, cool streams, lakes, rivers, reservoirs, and marshes (Epler et al. 2013).

***Paratanytarsustolucensis* Reiss, 1972: 62** [Mexico: Mexico State, Nevado de Toluca]. Andersen et al. (2000: 590). Endemic.

**Genus *Paratendipes* Kieffer, 1911**

A genus of nearly 40 named species that occur in the Afrotropical and Oriental regions and in the Holarctic realm (Qi et al. 2009). For South America there are only records of unnamed species (Roque et al. 2004; Trivinho-Strixino 2011). Larvae are found in lakes, ponds, small water bodies, bogs, and hot springs and in streams and rivers in soft sediments and sandy bottoms (Epler et al. 2013).

***Paratendipes* sp.**: Contreras-Ramos and Andersen (1999: 4) [Campeche State]; Vinogradova and Riss (2007: 33) [“Yucatan Peninsula”]; Bogan et al. (2014: 2726) [Sonora State]; Hamerlík et al. (2018: 217) [Yucatan State].

**Genus *Phaenopsectra* Kieffer, 1921**

A genus of more than ten named species that occur in all zoogeographical regions except the Antarctic, Oriental, and Australasian regions. The larvae mainly occur in sandy and muddy sediments of small standing and flowing waters, but also on submerged water plants and hard substrata (Epler et al. 2013).

***Phaenopsectra* sp.**: Navarrete-Salgado et al. (2004: 157) [México State]; Vinogradova and Riss (2007: 33) [“Yucatan Peninsula”].

**Genus *Polypedilum* Kieffer, 1912**

***Asheum* Sublette & Sublette, 1983, as subgenus**

Syn.: *Pedionomus* Sublette, 1964 (see Sæther and Sundal 1999).

The largest genus of Chironomidae, with > 500 described species that occur in all zoogeographical regions except Antarctica. Based on imaginal characters, eight subgenera were recognized by Sæther et al. (2010), namely *Tripedilum* Kieffer, 1921; *Polypedilum* s. str.; *Pentapedilum* Kieffer, 1913; *Tripodura* Townes, 1945; *Uresipedilum* Oyewo & Sæther, 1998; *Cerobregma* Sæther & Sundal, 1999; *Kribionympha* Kieffer, 1921; and *Probolum* Andersen & Sæther, 2010. However, the delimitation of the subgenera within *Polypedilum* was questioned by Yamamoto and Yamamoto (2015) and Cranston et al. (2016b). The position of Asheum is unclear but is usually treated as a subgenus within Polypedilum (see Pinho and Silva 2020). Larvae of *Polypedilum* occur in virtually all still and flowing waters, except in the Arctic and at high elevation. They are mostly found in sediments, mining water plants or specializing in plant-held waters (phytotelmata) (Epler et al. 2013).

**Polypedilum (Asheum) beckae (Sublette, 1964a: 137)** (*Pedionomus*) [USA: Louisiana]. Andersen et al. (2000: 590) [States of Campeche; Veracruz]; Contreras-Ramos et al. (2000: 25). NT, NE. Dominican Republic, Mexico, USA.

**Polypedilum (Asheum) curticaudatum Rempel, 1939: 214** [Brazil]. Vinogradova and Riss (2007: 33) (as: *Pedionomuscurticaudatus*) [“Yucatan Peninsula”]; Pinho and Silva (2020: 184). NT. Brazil, Mexico.

**Polypedilum (Polypedilum) purus Bidawid-Kafka, 1996: 216** [Brazil]. Vinogradova and Riss (2007: 33) [“Yucatan Peninsula”]. NT. Brazil, Mexico.

**Polypedilum (Tripodura) bacalar Vinogradova, 2008: 279** [Mexico: Quintana Roo State, Chetumal, Lake Bacalar]; Zhang et al. (2016: 41). Endemic.

**Polypedilum (Tripodura) rissi Vinogradova, 2008: 281** [Mexico: Yucatan State, Lake Punta Laguna]. Vinogradova (2008: 281) [Quinto Roo]; Zhang et al. (2016: 47). NT. Guatemala, Mexico

**Polypedilum (Tripodura) spiesi Vinogradova, 2008: 278** [Belize]. Vinogradova (2008: 278) [Quintana Roo State]; Zhang et al. (2016: 48). NT. Belize, Mexico.

**Polypedilum (Uresipedilum) pedatum Townes, 1945: 55** [USA: New York & Washington]. Andersen et al. (2000: 590) [Nuevo León State]. NE. Canada, Mexico, USA.

**Remarks.**Townes (1945: 55) described two subspecies, *P.pedatumpedatum* from New York and *P.pedatumexcelsius* from Washington. Andersen et al. (2000: 590) recorded the species as P. (Polypedilum) pedatum Townes, while Sæther and Oyewo (2008: 3) placed it in subgenusUresipedilum.

***Polypedilumrohneri* Vinogradova, 2008: 286** [Belize]. Vinogradova (2008: 286) [Yucatan State]. NT. Belize, Guatemala, Mexico.

**Remarks.** The species is not assigned to a subgenus. According to Vinogradova (2008: 288) it might deserve a separate subgenus.

**Genus *Pseudochironomus* Malloch, 1915**

A genus with at least 30 species distributed in the Neotropical, Nearctic, and Palaearctic regions. The Brazilian species have recently been treated by Shimabukuro et al. (2017) and Trivinho-Strixino and Shimabukuro (2018). The larvae inhabit sandy or gravelly littoral sediments, primarily in meso- or oligotrophic lakes or in large, slow flowing rivers (Epler et al. 2013).

***Pseudochironomusseipi* Andersen, 2023** [Mexico: Chiapas State, Chintul, Río Chintul]. NT. Costa Rica, Mexico.

**Genus *Rheotanytarsus* Thienemann & Bause, 1913**

A genus with ~ 100 species distributed in all zoogeographic regions except Antarctica. The Central American and Mexican species were reviewed by Kyerematen and Andersen (2002); the *Rheotanytarsuspellucidus* group was revised by Kyerematen et al. (2000). Larvae are rheobiontic, occurring in streams, large rivers, and the littoral of lakes where wave action simulates the action of flowing water (Epler et al. 2013).

***Rheotanytarsuscalakmulensis* Kyerematen & Andersen, 2002: 33** [Mexico: Campeche State, Calakmul Biosphere Reserve]. Endemic.

***Rheotanytarsuscontrerasi* Andersen & Sæther in Kyerematen et al., 2000: 166** [Mexico: Puebla State, Mpio. Progreso, Río San Juan]. Kyerematen et al. (2000: 166) [Nuevo León State]. Endemic.

***Rheotanytarsusfoliatus* Kyerematen & Andersen, 2002: 35** [Costa Rica]. Kyerematen and Andersen (2002: 35) [Nuevo León State]. NT. Costa Rica, Mexico.

***Rheotanytarsushanseni* Kyerematen & Andersen, 2002**: **42** [Mexico: Oaxaca State, Candelaria Loxiela]. Kyerematen and Andersen (2002: 42) [Morelos State]. Endemic.

***Rheotanytarsuskusii* Kyerematen & Andersen, 2002: 37** [Mexico: Nuevo León State, Allende, Río Ramos]. Endemic.

***Rheotanytarsusnuamae* Kyerematen & Andersen, 2002: 38** [Mexico: Nuevo León State, Allende, Río Ramos]. Endemic.

***Rheotanytarsusramirezae* Kyerematen & Andersen, 2002: 46** [Mexico: Nuevo León State, Santiago, Cola de Caballo]. Endemic.

**Genus *Saetheria* Jackson, 1977**

A genus of seven named species that occur in the Neotropical, Nearctic, and Palearctic regions (Orel 2014). Only unnamed larvae have so far been recorded from South America (Roque et al. 2004). Larvae inhabit sandy substrata of lakes and streams (Epler et al. 2013).

***Saetheria* sp.**: Vinogradova and Riss (2007: 33) [“Yucatan Peninsula”].

**Genus *Skutzia* Reiss, 1985**

A genus of six species that occur in the Neotropical, Nearctic, and Oriental regions. The genus was revised by Pinho et al. (2009a). The larvae are unknown. However, they can be expected to construct transportable cases of sand grains, small wood or plant remains, as seen in the larvae of other species in the subtribe Zavreliina.

***Skutziaquetzali* Pinho, Mendes & Andersen, 2009a: 204** [Mexico: Campeche State, Calakmul, Ejido Nuevo Becan, El Chorro]. NT. Mexico, Panama.

**Genus *Stempellina* Thienemann & Bause, 1913**

A genus of at least 20 species that occur in all zoogeographic regions except Antarctica. The larvae construct long, curved, tapered, transportable cases of fine sand and silt. They are eurytopic, occurring in springs, streams, larger rivers, lakes, brackish water, moorland pools, and in thermal springs (Epler et al. 2013).

***Stempellina* sp.**: Contreras-Ramos and Andersen (1999: 4) [Campeche State]; Vinogradova and Riss (2007: 34) [“Yucatan Peninsula”]; Bogan et al. (2014: 2726) [Sonora State].

**Genus *Stempellinella* Brundin, 1947**

A genus of ~ 20 described species that occur in all zoogeographical regions except Antarctica. The larvae construct straight, transportable cases of fine sand and silt, often speckled with detritus; they occur in unpolluted springs and small streams as well as in lakes (Epler et al. 2013).

***Stempellinella* sp.**: Vinogradova and Riss (2007: 34) [“Yucatan Peninsula”]; Bogan et al. (2014: 2726) [Sonora State].

**Genus *Stenochironomus* Kieffer, 1919**

A genus of > 100 described species that occur in all zoogeographic regions except Antarctica. The genus was revised by Borkent (1984). South American species were treated by Dantas et al. (2016). Larvae are obligate miners in living or dead vegetation including woody parts of plants, in both lentic and lotic situations (Epler et al. 2013).

***Stenochironomusleptopus* Kieffer, 1906**: **19** [St. Vincent]. Andersen et al. (2000: 590) [Mexico, without specific locality]. NT. Costa Rica, Dominica, Ecuador, Guatemala, Mexico, St. Vincent.

**Genus *Sublettea* Roback, 1975**

A small genus with four species distributed in the Neotropical, Nearctic, and Oriental regions (Ashe et al. 1987). The larvae occur in flowing waters including cool, clean, fast flowing, temperate streams and warm, tropical rivers and streams (Epler et al. 2013). The only known larva construct soft, non-transportable cases of fine granules and silk that are attached to the substrate (Roback 1975).

***Sublettea* sp.**: Vinogradova and Riss (2007: 34) [“Yucatan Peninsula”].

**Genus *Tanytarsus* Wulp, 1874**

Syn.: *Nimbocera* Reiss, 1972 (see Sanseverino et al. 2010).

Syn.: *Caladomyia* Säwedal, 1981 (see Lin et al. 2018).

A species-rich genus with > 350 described species that occur in all zoogeographic regions except Antarctica. A molecular phylogeny of the genus was presented by Lin et al. (2018), placing *Caladomyia* as a junior synonym of *Tanytarsus*. The larvae are found in all types of freshwaters, with some marine, and at least one terrestrial species. The freshwater species usually construct long, soft tubes that are fixed to the bottom substrate (Epler et al. 2013).

***Tanytarsushastatus* Sublette & Sasa, 1994: 56** [Guatemala]. Andersen et al. (2000) [Sinaloa State]; Vinogradova and Riss (2007) [“Yucatan Peninsula”]. European Bioinformatics Institute (2022). NT, NE. Brazil, Costa Rica, Ecuador, Guatemala, Mexico, Panama, Peru, USA, Venezuela.

***Tanytarsuspistra*** (**Sublette & Sasa, 1994: 54)** (*Caladomyia*) [Guatemala]. Vinogradova and Riss (2007: 32) [“Yucatan Peninsula”]. NT, NE. Guatemala, Mexico, USA (Lathrop and Mulla 1995).

**Genus *Tribelos* Townes, 1945**

A genus with less than 10 named species distributed mainly in the Nearctic and Palaearctic regions. The genus is also recorded from the Neotropical region (Trivinho-Strixino et al. 2000). The larvae occur in littoral sediments of small to large water bodies (Epler et al. 2013).

***Tribelos* sp.**: Contreras-Ramos and Andersen (1999: 4) [Campeche State].

**Genus *Xenochironomus* Kieffer, 1921**

A genus with ~ 20 species distributed in the Neotropical, Nearctic, Palaearctic, Oriental, and Australasian regions. The genus was revised by Fusari et al. (2013). The larvae of almost all species are obligate miners in freshwater sponges in standing and flowing waters (Epler et al. 2013).

***Xenochironomus* sp.**: Contreras-Ramos and Andersen (1999: 4) [Campeche State]; Vinogradova and Riss (2007: 33) [“Yucatan Peninsula”].

**Genus *Xestochironomus* Sublette & Wirth, 1972**

A genus of more than ten described species that occur only in the Neotropical and Nearctic regions (Pinho and Souza 2013; Bello-González et al. 2016). Known larvae are miners in immersed wood in running waters (Epler et al. 2013).

***Xestochironomuslatilobus* Borkent, 1984: 29** [Venezuela]. Andersen et al. (2000: 590) [Campeche State]; Contreras-Ramos et al. (2000: 26). NT. Costa Rica, Mexico, Venezuela.

**Genus *Zavreliella* Kieffer, 1920**

A genus with ~ 15 species; according to Fusari et al. (2017), 13 of these are known from tropical South America. The genus was revised by Reiss (1990). Larvae build transportable cases and move among submerged vegetation in standing water, but can also be found in sediments in flowing waters (Epler et al. 2013).

***Zavreliellalongiseta* Reiss, 1990: 112** [Brazil]. Contreras-Ramos and Andersen (1999: 4, as *Omisus* sp.) [Campeche State]; Contreras-Ramos et al. (2000: 26, as *Omisus* sp.); Vinogradova and Riss (2007: 33) [“Yucatan Peninsula”]. NT. Brazil, Costa Rica, Mexico, Panama.

**Remarks.** The genus *Omisus* Townes, 1945 was recorded from Campeche State by Contreras-Ramos and Andersen (1999) and Contreras-Ramos et al. (2000). However, this record is incorrect. At closer examination the specimens belong to *Zavreliellalongiseta* Reiss, 1990, a species that lacks dark spots in the wing and has a second, strong, curved spur on the hid tibia. The generic diagnosis given by Reiss (1990) should thus be amended accordingly.

#### Subfamily Diamesinae

**Genus *Diamesa* Meigen, 1835**

A genus of > 100 named species distributed in the Nearctic, Palaearctic, Afrotropical, and Oriental regions. Larvae of *Diamesa* are generally adapted to cool waters, inhabiting flowing water, springs, and to a lesser extent shallow still water and the hygropetric zone; they can be dominant in the kryon zone of glacier fed streams (Sæther and Andersen 2013a).

***Diamesamexicana* Serra-Tosio, 1977: 100** [Mexico: Mexico State, Lake Nevado de Toluca]. Andersen et al. (2000: 589); Ashe and O’Connor (2009: 281). Endemic.

***Diamesareissi* Serra-Tosio, 1977: 99** [Mexico: Mexico State, Lake Nevado de Toluca]. Andersen et al. (2000: 589); Ashe and O’Connor (2009: 283). Endemic.

**Genus *Pseudokiefferiella* Zavřel, 1941**

The only included species, *Pseudokeifferiellaparva* (Edwards, 1932), is distributed in the Nearctic and Palaearctic regions. The larvae inhabit small streams and the hygropetric zone (Sæther and Andersen 2013a).

***Pseudokiefferiella* sp.**: Bogan et al. (2014: 2726) [Sonora State].

#### Subfamily Orthocladiinae

Syn.: Prodiamesinae (see Lin et al. 2022).

**Genus *Allocladius* Kieffer, 1913**

A genus of 25 named species that occur in all zoogeographical regions, except Antarctica and Oceania. Andersen et al. (2010) reviewed the South American species; a revision of the genus was given by Ferrington and Sæther (2011). The larvae of *Allocladius* appear to be truly aquatic, as they have been found in ponds, rivers, and streams, including the shores of brackish water bodies and salt marshes, but some are probably able to survive in moist sandy substrata (Andersen et al. 2013b).

***Allocladiusnanseni* (Kieffer, 1926: 82)** (*Camptocladius*) [Canada]. Ferrington and Sæther (2011: 66) [Mexico State]; Ashe and O’Connor (2012a: 118). NE, PA. Widespread.

**Genus *Antillocladius* Sæther, 1981**

A genus of 30 named species that occur mostly in the Neotropical region, but are also found in the Nearctic, Palaearctic, and Oriental regions (Ashe and O’Connor 2012a; Andersen and Hagenlund 2017). The genus was reviewed by Mendes et al. (2004, 2011) and Mendes and Andersen (2008). Known larvae from South America appear to be terrestrial or semi-terrestrial as they have been collected in moss and lichens on stones and tree trunks; a North American species has been found in seeps near streams and impoundments (Mendes et al. 2004; Andersen et al. 2013b).

***Antillocladiusarcuatus* Sæther, 1982: 474** [USA: South Carolina]. Mendes et al. (2004: 29) [Nuevo León State]; Mendes and Andersen (2008: 21); Ashe and O’Connor (2012a: 121). NT, NE. Brazil, Mexico, USA, Venezuela.

***Antillocladiuscalakmulensis* Mendes, Andersen & Sæther, 2004: 32** [Mexico: Campeche State, Calakmul Biosphere Reserve]. Mendes and Andersen (2008: 28); Ashe and O’Connor (2012a: 122); Admin (2022). Endemic.

***Antillocladiusherradurus* Mendes, Andersen & Sæther, 2004: 39** [Mexico: Campeche State, Calakmul Biosphere Reserve]. Mendes and Andersen (2008: 33); Ashe and O’Connor (2012a: 122); Admin (2022). Endemic.

***Antillocladiuspluspilalus* Sæther, 1982: 474** [USA: South Carolina]. Mendes et al. (2004: 48) [Campeche State]; Mendes and Andersen (2008: 36); Ashe and O’Connor (2012a: 122). NT, NE. Ecuador, Mexico, Nicaragua, USA.

***Antillocladiuszempoalensis* Mendes, Andersen & Sæther, 2004: 57** [Mexico: Morelos State, Lagunas de Zempoala National Park]. Mendes and Andersen (2008: 41); Ashe and O’Connor (2012a: 124). Endemic.

**Genus *Bryophaenocladius* Thienemann, 1934**

A species-rich genus with ~ 120 named species that occur in all zoogeographic regions, except Antarctica and Oceania. Neotropical and Mexican species were reviewed by Wang et al. (2006). The larvae of most species are terrestrial or semi-terrestrial, but a few are aquatic (Andersen et al. 2013b).

***Bryophaenocladiusdigitatus* Sæther, 1973: 55** [USA: South Dakota]. Wang et al. (2006: 23) [Campeche State]; Ashe and O’Connor (2012a: 141). NE. Mexico, USA.

***Bryophaenocladiushumerosus* Wang, Andersen & Sæther, 2006: 26** [Mexico: Morelos State, Lagunas de Zempoala National Park]. Ashe and O’Connor (2012a: 144); Admin (2022). Endemic.

***Bryophaenocladiuspichinensis* Wang, Andersen & Sæther, 2006: 28** [Ecuador]. Wang et al. (2006: 28) [States of Nuevo León; Puebla]; Ashe and O’Connor (2012a: 150); Admin (2022). NT. Ecuador, Mexico.

***Bryophaenocladiussimplex* Wang, Andersen & Sæther, 2006: 30** [Mexico: Nuevo León State, Allende, Río Ramos]. Wang et al. (2006: 30) [Nuevo León State, Santiago]; Ashe and O’Connor (2012a: 152); Admin (2022). Endemic.

**Genus *Cardiocladius* Kieffer, 1912**

A genus of 20 named species that occur in all zoogeographic regions except Antarctica and Oceania. The Neotropical species were reviewed by Andersen et al. (2016). The larvae live in fast-flowing waters and are often associated with the immature stages of blackflies (Simuliidae), on which they are reported to be predaceous (Andersen et al. 2013b).

***Cardiocladiusmoreloensis* Andersen, Hagenlund & Pinho, 2016: 277** [Mexico: Morelos State, Estación Ceamish]. Endemic.

**Genus *Clunio* Haliday, 1855**

A genus of 25 described species that occur in all zoogeographic regions except Antarctica. The larvae are marine and believed to be omnivorous, feeding on algae and dead or dying animals (Andersen et al. 2013b).

***Clunio* sp.**: Sotelo-Casas et al. (2014: 17) [Nayarit State: Marieta Islands].

**Genus *Corynoneura* Winnertz, 1846**

A genus of ~ 100 named species that occur in all zoogeographic regions except Antarctica. A review of the Neotropical species was given by Wiedenbrug et al. (2012). Larvae occur in virtually all types of aquatic habitats, from standing waters to fast-flowing streams (Andersen et al. 2013b).

***Corynoneurazempoala* Wiedenbrug, Lamas & Trivinho-Strixino, 2012: 55.** [Mexico: Morelos State, Parque Nacional Lagunas de Zempoala]. Endemic.

**Genus *Cricotopus* Wulp, 1874**

Syn.: *Paratrichocladius* Santos Abreu, 1918 (see Cranston and Krosh 2015)

A genus of ~ 270 named species that occur in all zoogeographic regions except Antarctica. Seven subgenera are recognized, namely *Cricotopus* s. str.; *Isocladius* Kieffer, 1909; *Maurius* Lehmann, 1981; *Nostocladius* Ashe & Murray, 1980; *Oliveiriella* Wiedenbrug & Fittkau, 1997; *Paratrichocladius* Santos Abreu, 1918; and *Pseudocricotopus* Nishida, 1987 (see Ashe and O’Connor 2012a; Andersen et al. 2013b; Cranston and Krosch 2015). Larvae inhabit all types of freshwaters including saline coastal waters. They are frequently associated with aquatic plants, including algae, and some mine living parts of aquatic macrophytes (Andersen et al. 2013b).

**Cricotopus (Cricotopus) bicinctus (Meigen, 1818: 41)** (*Chironomus*) [Austria]. Andersen et al. (2000: 589) [States of Mexico; Guerrero; Sinaloa]; Ashe and O’Connor (2012a: 209). NT, NE, PA, OR, OC. Widespread.

**Cricotopus (Isocladius) sylvestris (Fabricius, 1794: 252)** (*Tipula*) [Germany]. Andersen et al. (2000: 589) [States of Mexico; Guanajuato]; Ashe and O’Connor (2012a: 245). NT, NE, PA. Widespread.

**Cricotopus (Cricotopus) triannulatus (Macquart, 1826: 202)** (*Chironomus*) [France]. Andersen et al. (2000: 589) [Puebla State]; Ashe and O’Connor (2012a: 231); Alcocer et al. (2016: 411). NE, PA. Widespread.

**Genus *Diplosmittia* Sæther, 1981**

A genus of 10 named species distributed in the Neotropical and Nearctic regions. A review of the genus was provided by Pinho et al. (2009b). Wiedenbrug and Silva (2016) added a species from the Dominican Republic. The immatures are unknown.

***Diplosmittiaharrisoni* Sæther, 1981: 30** [St. Lucia]. Pinho et al. (2009b: 177) [Campeche State]; Ashe and O’Connor (2012a: 262). NT. Costa Rica, Mexico, St. Lucia, St. Vincent, Venezuela.

**Genus *Gravatamberus* Mendes & Andersen, 2008**

A genus with five named species endemic to the Neotropical region. Larvae have been found in bromeliads (Mendes and Andersen 2008).

***Gravatamberuscurtus* Mendes & Andersen, 2008: 45** [Mexico: Campeche State, Calakmul Biosphere Reserve]. Ashe & O’Connor (2012a: 293); Admin (2022). NT. Costa Rica, Mexico.

**Remarks.**Epler (2017) recorded *Gravatamberusguatemaltecus* Mendes & Andersen, 2008 from Zurquí de Moravia in Costa Rica and commented on the variation in *G.curtus*.

**Genus *Limnophyes* Eaton, 1875**

A genus of > 90 named species that occur in all zoogeographic regions except Oceania. Sæther (1990a, b) revised the Holarctic, Afrotropical, and Neotropical species of the genus. The larvae are eurytopic, including aquatic, semiterrestrial and terrestrial habitats (Andersen et al. 2013b).

***Limnophyes* sp.**: Andersen et al. (2000: 591) [Puebla State].

**Genus *Lopescladius* Oliveira, 1967**

A genus with eight named species from the Neotropical and Nearctic regions. Two subgenera are recognized, namely *Lopescladius* s. str. and *Cordiella* Coffman & Roback, 1984 (see Ashe and O’Connor 2012a). South American species of Lopescladius (Cordiella) were described by Hagenlund et al. (2010). Larvae inhabit streams with sandy sediments (Trivinho-Strixino 2011).

**Lopescladius (Lopescladius) verruculosus Sæther, 1983: 289** [Mexico: Michoacán State, Tocuman]. Andersen et al. (2000: 589); Ashe and O’Connor (2012a: 365). NE. Mexico, USA.

**Genus *Mesosmittia* Brundin, 1956**

A genus of 18 named species that occur in the Neotropical, Nearctic, Palaearctic, Afrotropical, and Oriental regions. The Neotropical and Mexican species were reviewed by Andersen and Mendes (2002b). The immatures are likely terrestrial (Andersen et al. 2013b).

***Mesosmittiaacutistylus* Sæther, 1986: 43** [USA: New Mexico]. Andersen & Mendes (2002b: 143) [Campeche State]; Ashe and O’Connor (2012a: 369). NE. Mexico, USA.

***Mesosmittiaannae* Andersen & Mendes, 2002b: 143** [Guatemala]. Andersen and Mendes (2002b: 143) [Campeche State]; Ashe and O’Connor (2012a: 369); Admin (2022). NT. Guatemala, Mexico.

***Mesosmittiaguanajensis* Andersen & Mendes, 2002b: 147** [Mexico: Guanajuato State, Acámbaro]. Ashe and O’Connor (2012a: 370); Admin (2022). Endemic.

***Mesosmittialobiga* Sæther, 1986: 45** [USA: New Mexico]. Andersen and Mendes (2002b: 150) [States of Guanajuato; Nuevo León]; Ashe and O’Connor (2012a: 370). NT, NE. Mexico, Puerto Rico, USA.

***Mesosmittiapatrihortae* Sæther, 1986: 47** [USA: South Carolina]. Andersen and Mendes (2002b: 150) [States of Campeche; Nuevo León; Veracruz]; Ashe and O’Connor (2012a: 371). NT, NE, PA, AF. Widespread.

**Remarks.** Based on material collected in Zurquí, Costa Rica, Epler (2017) could not separate *M.truncata* from *M.patrihortae* Sæther, 1986, and considered *M.truncata* to be a junior synonym of *M.patrihortae*.

***Mesosmittiaprolixa* Sæther, 1986: 48** [USA: Kansas]. Andersen and Mendes (2002b: 150) [States of Campeche; Nuevo León]; Ashe and O’Connor (2012a: 371). NE. Mexico, USA.

***Mesosmittiatora* Sæther, 1986: 50** [USA: South Dakota]. Andersen and Mendes (2002b: 150) [Nuevo León State]; Ashe and O’Connor (2012a: 371). NE. Mexico, USA.

**Genus *Metriocnemus* Wulp, 1874**

A genus of 75 named species that occur in all zoogeographic regions except Antarctica and Oceania. Three subgenera are recognized, namely *Metriocnemus* s. str.; *Crymaleomyia* Ashe & O’Connor, 2000; and *Inermipupa* Langton & Cobo, 1997 (see Ashe and O’Connor 2012a). A review of the genus was given by Sæther (1995). Larvae occur in mosses, phytotelmata, springs, ditches, streams and lakes and a few species are hygropetric (Andersen et al. 2013b).

***Metriocnemus* sp.**: Andersen et al. (2000: 591) [Nuevo León State].

**Genus *Nanocladius* Kieffer, 1913**

A genus of 37 named species that occur in all zoogeographic regions except Antarctica. Two subgenera are recognized, namely *Nanocladius* s. str., and *Plecopteracoluthus* Steffan, 1965 (see Ashe and O’Connor 2012a). Neotropical species were treated by Wiedenbrug and Silva (2013). Larvae occur in streams, rivers, lakes, and ponds and some are symphoretic on immature Megaloptera and Ephemeroptera (Andersen et al. 2013b).

***Nanocladius* sp.**: Contreras-Ramos and Andersen (1999: 4) [Campeche State]; Vinogradova and Riss (2007: 33) [“Yucatan Peninsula”].

**Genus *Onconeura* Andersen & Sæther, 2005**

A genus of eight named species that occur in the Neotropical and Nearctic regions. A review of the genus was given by Wiedenbrug et al. (2009), and a cladistic analysis of the genus was given by Donato et al. (2012). The larvae inhabit streams and rivers (Andersen et al. 2013b).

***Onconeurasemifimbriata* (Sæther, 1981: 32)** (*Thienemanniella*) [St. Vincent]. Andersen and Sæther (2005: 13) [Nuevo León State]; Wiedenbrug et al. (2009: 13); Ashe and O’Connor (2012a: 408). NT. Brazil, Costa Rica, Guatemala, Mexico, St. Vincent.

**Genus *Orthocladius* Wulp, 1874**

A genus of ~ 150 named species that occur in the Nearctic, Palaearctic, Afrotropical, and Oriental regions. Six subgenera are recognized, *Orthocladius* s. str., *Eudactylocladius* Thienemann, 1935; *Euorthocladius* Thienemann, 1935; *Mesorthocladius* Sæther, 2005; *Pogonocladius* Brundin, 1956; and *Symposiocladius* Cranston, 1982 (see Ashe and O’Connor 2012a). The genus is recorded from South America based on unnamed larvae from Argentina belonging to the subgenus Eudactylocladius (Wais 1987). The larvae inhabit all types of flowing waters, lakes, ponds, swamps, and moist earth; some species also mine submerged wood (Andersen et al. 2013b).

**Orthocladius (Euorthocladius) sp.**: Andersen et al. (2000: 591) [Mexico State].

**Orthocladius (Orthocladius) sp.**: Andersen et al. (2000: 591) [Mexico State].

**Genus *Paralimnophyes* Brundin, 1956**

A genus of five named species that occur in the Nearctic, Palaearctic, Oriental, and Australasian regions. The only species with described larvae inhabits eutrophic lowland pools and ditches (Andersen et al. 2013b).

***Paralimnophyes* sp.**: Andersen et al. (2000: 591) [Puebla State]; Contreras-Ramos et al. (2000: 25) [Campeche State].

**Genus *Parametriocnemus* Goetghbuer, 1932**

A genus of 35 named species that occur in all zoogeographic regions except Antarctica and the Neotropical region. The genus is recorded from South America based on unnamed larvae from Brazil, Colombia, Peru, and Venezuela (Roback and Coffman 1983; Ospina-Torres et al. 1999; Trivinho-Strixino 2011). Larvae of *Parametriocnemus* are found in springs and in relatively fast flowing cold streams and rivers (Andersen et al. 2013b).

***Parametriocnemus* sp.**: Contreras-Ramos and Andersen (1999: 4) [Campeche State]; Bogan et al. (2014: 2726) [Sonora State].

**Genus *Paratrichocladius* Santos Abreu, 1918**

See: *Cricotopus* Wulp, 1874.

**Genus *Prodiamesa* Kieffer, 1906**

A genus of six named species distributed in the Nearctic and Palaearctic regions. Larvae of *Prodiamesa* occur in springs, streams, rivers, ponds, and the littoral zone in lakes (Sæther and Andersen 2013b).

***Prodiamesa* sp.**: Granados-Ramírez et al. (2017: 45) [Morelos State].

**Genus *Psectrocladius* Kieffer, 1906**

A genus with > 60 named species that occur in all zoogeographic regions, except Antartica, Australasia, Oceania, and the Neotropical region. Four subgenera are recognized, namely *Psectrocladius* s. str.; *Allopsectrocladius* Wülker, 1956; *Mesopsectrocladius* Laville, 1972; and *Monopsectrocladius* Wülker, 1956 (see Ashe and O’Connor 2012a). The only record from South America is an unnamed larval morphotype from the Peruvian Amazon belonging to subgenus Psectrocladius (Roback 1966). The larvae are eurytopic (Andersen et al. 2013b).

***Psectrocladius* sp.**: Andersen et al. (2000: 591) [Puebla State]; Bogan et al. (2014: 2726) [Sonora State].

**Genus *Pseudosmittia* Edwards, 1932**

A genus of > 100 described species that occur in all zoogeographic regions, except Antarctica. Andersen et al. (2010) reviewed the Neotropical species, and a revision of the genus was given by Ferrington and Sæther (2011). Most larvae appear to be semiterrestrial to semiaquatic (Andersen et al. 2013b).

***Pseudosmittiaforcipata* (Goetghebuer, 1921; 87)** (*Camptocladius*) [Belgium]. Andersen et al. (2010: 39) [States of Campeche; Nuevo León]; Ferrington & Sæther (2011: 297); Ashe and O’Connor (2012a: 545). NT, NE, PA, OR. Widespread.

***Pseudosmittiainvirgata* Andersen, Sæther & Mendes, 2010: 43** [Mexico: Campeche State, Calakmul Biosphere Reserve]. Ferrington and Sæther (2011: 288); Ashe and O’Connor (2012a: 547). Endemic.

***Pseudosmittiajoaquimvenancioi* (Messias & Oliveira, 2000: 189)** (*Bryophaenocladius*) [Brazil]. Wang et al. (2006: 19); Andersen et al. (2010: 45) [States of Campeche; Veracruz]; Ferrington and Sæther (2011: 184); Ashe and O’Connor (2012a: 547). NT. Brazil, Costa Rica, Mexico, Nicaragua, St. Lucia, St. Vincent, Venezuela.

**Genus *Rheocricotopus* Brundin, 1956**

A genus of ~ 75 described species that occur in all zoogeographic regions except Antarctica and Oceania. Two subgenera are recognized, namely *Rheocricotopus* s. str., and *Psilocricotopus* Sæther, 1986 (see Ashe and O’Connor 2012a). The first named species from the Neotropical region, Rheocricotopus (Psilocricotopus) sirventorum Andersen & Mendes, was recently described from Brazil by Andersen and Mendes (2012). Larvae are rheophilic, living on plants and stones in streams and rivers, and are rarely found in the littoral zone of lakes (Andersen et al. 2013b).

***Rheocricotopus* sp.**: Andersen et al. (2000: 589) [Mexico State].

**Genus *Smittia* Holmgren, 1869**

A species-rich genus with > 80 named species that occur in all zoogeographic regions except Antarctica. Most larvae are terrestrial, occurring in damp soil, but at least one species is aquatic (Andersen et al. 2013b).

***Smittia* sp.**: Andersen et al. (2000: 591) [Baja California Sur State]; Hamerlík et al. (2018: 217) [Yucatán State].

**Genus *Synorthocladius* Thienemann, 1935**

A genus of eight named species that occur in all zoogeographic regions except Antarctica. The larvae inhabit springs, small to large bodies of flowing water and small bodies or shallow parts of still water (Andersen et al. 2013b).

***Synorthocladiussemivirens* (Keiffer, 1909: 48)** (*Dactylocladius*) [Germany]. Andersen et al. (2000: 589) [Mexico State]; Ashe and O’Connor (2012a: 610). NE, PA, OR. Widespread.

**Genus *Thienemanniella* Kieffer, 1911**

A genus of ~ 55 named species that occur in all zoogeographic regions except Antarctica. The Neotropical species were reviewed by Wiedenbrug et al. (2013). The larvae occur in most lotic habitats, from fast-flowing streams to slow-flowing ditches and rivers (Andersen et al. 2013b).

***Thienemanniella* sp.**: Contreras-Ramos and Andersen (1999: 4) [Campeche State]; Bogan et al. (2014: 2726) [Sonora State]; Granados Ramírez et al. (2017: 45) [States of Mexico; Morelos].

#### Subfamily Prodiamesinae

See Subfamily Orthocladiinae.

#### Subfamily Tanypodinae

**Genus *Ablabesmyia* Johannsen, 1905**

A genus of nearly 100 described species that occur in all zoogeographic regions, except Antarctica; it is currently the most speciose genus in Tanypodinae. Four subgenera, *Ablabesmyia* s. str., *Asaya* Roback, 1985, *Karelia* Roback, 1971, and *Sartaia* Roback, 1983 are recognized (see Ashe and O’Connor 2009). Most Neotropical species probably belong in *Ablabesmyia* s. str., but as pointed out by several authors, many South American species cannot be assigned to a subgenus with certainty, as there are inconsistencies in the establishment of these groups (see Neubern et al. 2013). Many of the recently described species are thus not assigned to a subgenus. The Neotropical species were reviewed by Neubern et al. (2013). The larvae occur in a wide variety of habitats, including small and large standing and flowing waters from cold temperate to warm tropical climate zones (Cranston and Epler 2013).

**Ablabesmyia (Karelia) cinctipes (Johannsen, 1946: 271)** (*Pentaneura*) [USA: Florida]. Andersen et al. (2000: 589) [States of Chiapas; Guerrero]; Vinogradova and Riss (2007: 33) [“Yucatan Peninsula”]; Ashe and O’Connor (2009: 121); Cranston and Epler (2013: 62). NT, NE. Bahamas, Belize, Guatemala, Mexico, St. Vincent, USA.

**Genus *Alotanypus* Roback, 1971**

A genus of 11 described species distributed in the Neotropical, Nearctic, Palaearctic, and Australasian regions. Larvae occur in both standing and flowing waters and appear to tolerate a broad range of conditions including very acid waters (Cranston and Epler 2013).

***Alotanypus* sp.**: Andersen et al. (2000: 591) [Nuevo León State].

**Genus *Apsectrotanypus* Fittkau, 1962**

A genus of seven named species that occur in all zoogeographic regions except Antarctica and Oceania. In South America unnamed species are recorded from Argentina and Colombia (Donato et al. 2008b; Ruiz-Moreno et al. 2000; Spies and Reiss 1996). The larvae inhabit small, cool, flowing waters (Cranston and Epler 2013).

***Apsectrotanypus* sp.**: Bogan et al. (2014: 2725) [Sonora State].

**Genus *Clinotanypus* Kieffer, 1913**

A genus of ~ 45 described species that occur in all zoogeographic regions, except Antarctica. Two subgenera are recognized, *Clinotanypus* s. str. and *Aponteus* Roback, 1971 (see Ashe and O’Connor 2009.) The Neotropical species were reviewed by Neubern et al. (2014). The larvae prefer soft sediments in shallow, warm water bodies including ponds, lakes and slow-flowing streams and rivers (Cranston and Epler 2013).

***Clinotanypus* sp.**: Contreras-Ramos and Andersen (1999: 4) [Campeche State].

**Genus *Coelotanypus* Kieffer, 1913**

A genus of ~ 20 described species that occur in the Neotropical, Nearctic, Afrotropical, and Australasian regions. A key to the males of the Neotropical species was given by Paggi and Zilli (2018). The larvae inhabit benthic sediments of lakes, including artificial impoundments, slow flowing reaches of rivers and old riverbeds (Cranston and Epler 2013). The genus can be very abundant in Amazonian flood-plain lakes and in wetlands in southern Brazil (Fonseca Leal et al. 2004; Panatta et al. 2007).

***Coelotanypusatus* Roback, 1971: 37** [USA: Texas]. Andersen et al. (2000: 589) [Mexico, without specific locality]; Ashe and O’Connor (2009: 140). NT, NE. Mexico, Puerto Rico, USA.

***Coelotanypusconcinnus* (Coquillett, 1895: 308)** (*Tanypus*) [USA: Texas]. Andersen et al. (2000: 589) [Sonora State]; Ashe and O’Connor (2009: 141). NT, NE. Costa Rica, Mexico, Nicaragua, Puerto Rico, USA.

***Coelotanypusnaelis* Roback, 1963: 170** [Surinam]. Andersen et al. (2000: 589) [Veracruz State]; Ashe and O’Connor (2009: 142). NT, NE. Mexico, Panama, Surinam, USA.

***Coelotanypusolmecus* Roback, 1965: 33** [Mexico: Veracruz State]. Andersen et al. (2000: 589); Ashe and O’Connor (2009: 142). NT. Mexico, Nicaragua.

***Coelotanypusscapularis* (Loew, 1866: 2)** (*Tanypus*) [USA: Washington]. Andersen et al. (2000: 589) [Mexico, without specific locality]; Ashe and O’Connor (2009: 142). NT, NE. Canada, Mexico, Panama, USA.

***Coelotanypustoltecus* Roback, 1965: 32** [Mexico: Veracruz State]. Andersen et al. (2000: 589); Ashe and O’Connor (2009: 142). Endemic.

***Coelotanypustricolor* (Loew, 1861: 309)** (*Tanypus*) [USA: New York]. Andersen et al. (2000: 589) [Veracruz State]; Ashe and O’Connor (2009: 143). NT, NE. Costa Rica, Mexico, USA.

**Genus *Djalmabatista* Fittkau, 1968**

A genus of 15 described species that occur in all zoogeographic regions except Antarctica and Oceania. The larvae appear to prefer low alkalinity to weakly acid waters, and may be found in lakes, ponds, springs, large and small rivers, as well as in temperate to tropical lentic and lotic depositional habitats (Cranston and Epler 2013).

***Djalmabatistapulchra* (Johannsen, 1908: 273)** (*Protenthes*) [USA: New York]. Andersen et al. (2000: 589) [States of Chiapas; Guerrero]; Ashe and O’Connor (2009: 155). NT, NE. Argentina ( Oca et al. 2020), Bahamas (Anderson et al. 2014), Brazil, Canada, Costa Rica, Guatemala, Mexico, Nicaragua, USA.

**Genus *Fittkauimyia* Karunakaran, 1969**

A genus of eight named species that occur in all zoogeographic regions except Antarctica and Oceania. The larvae inhabit rivers and the littoral zone of lakes, generally in tropical and subtropical regions (Cranston and Epler 2013).

***Fittkauimyia* sp.**: Andersen et al. (2000: 591) [States of Campeche; Nuevo León]; Contreras-Ramos and Andersen (1999: 4); Contreras-Ramos et al. (2000: 25); Vinogradova and Riss (2007: 33) [“Yucatan Peninsula”]; Bogan et al. (2014: 2725) [Sonora State]; Hamerlík et al. (2018: 217) [States of Quintana Roo; Yucatán].

**Genus *Labrundinia* Fittkau, 1962**

A genus of ~ 40 named species distributed in the Neotropical, Nearctic, Palaearctic, and Oriental regions. The genus was revised by Silva et al. (2014). The larvae live in small, standing water bodies as well as in streams and rivers (Cranston and Epler 2013).

***Labrundiniafosteri* Roback, 1987: 2018** [Colombia]. Vinogradova and Riss (2007 33) [“Yucatan Peninsula”]; Ashe and O’Connor (2009: 164); Silva et al. (2014: 44). NT. Colombia, Mexico.

***Labrundinialongipalpis* (Goetghebuer, 1921: 66)** (*Tanypus*) [Belgium].

Syn.: *Labrundiniamaculata* Roback, 1971: 278 [USA: California] (Silva et al. 2011: 294).

Andersen et al. (2000: 589) [Coahuila State]; Ashe and O’Connor (2009: 165, 2012b: 127) [Michoacán State]; Silva et al. (2011: 295, 2014: 67). NT, NE, PA. Widespread.

***Labrundiniapilosella* (Loew, 1866: 5)** (*Tanypus*) [USA: District Columbia]. Andersen et al. (2000: 589) [Puebla State]; Ashe and O’Connor (2009: 166); Silva et al. (2014: 127). NT, NE. Canada, Guatemala, Honduras, Mexico, Puerto Rico, Trinidad and Tobago, USA, Venezuela.

**Genus *Larsia* Fittkau, 1962**

A genus of ~ 30 named species that occur in all zoogeographic regions except Antarctica. Neubern and Silva (2011) described two new species from the Neotropical region and presented a checklist of the *Larsia* species of the world. In the Southern Hemisphere the larvae are associated with both lotic and lentic warm waters (Cranston and Epler 2013).

***Larsiaplanensis* (Johannsen, 1946: 284)** (*Pentaneura*) [USA: Texas]. Andersen et al. (2000: 589) [Mexico City and States of Morelos; Oaxaca; Veracruz]; Ashe and O’Connor (2009: 169). NT, NE, OC. Canada, Guatemala, Hawaiian Islands, Mexico, USA.

**Genus *Natarsia* Fittkau, 1962**

A genus of six named species distributed in the Nearctic, Palaearctic, and Oriental regions. The larvae of the North American species live in small running waters, perhaps favoring cool water. European species inhabit streams, springs, and the littoral zone of montane or northern lakes and show hygropetric behavior in small, standing waters (Cranston and Epler 2013).

***Natarsia* sp.**: Vinogradova and Riss (2007: 33) [“Yucatan Peninsula”].

**Genus *Nilotanypus* Kieffer, 1923**

A genus of 11 named species distributed in all zoogeographical regions except Antarctica and Oceania. Andersen and Pinho (2019) recently described two new species of *Nilotanypus* from Brazil. The larvae inhabit flowing waters, especially areas with sandy beds (Cranston and Epler 2013).

***Nilotanypus* sp.**: Contreras-Ramos and Andersen (1999: 4) [Campeche]; Vinogradova and Riss (2007: 33) [“Yucatan Peninsula”].

**Genus *Paramerina* Fittkau, 1962**

See: *Zavrelimyia* Fittkau, 1962.

**Genus *Pentaneura* Philippi, 1866**

A genus of eight named species distributed in the Neotropical and Nearctic regions. Silva and Ferrington (2018) recently reviewed the Neotropical species. The larvae inhabit a variety of aquatic systems, from small streams and ponds to lakes and bays, occasionally the larvae live in shallow water flowing over bedrock covered with moss, algae, and detritus (Silva and Ferrington 2018).

***Pentaneurainconspicua* (Malloch, 1915: 371)** (*Tanypus*) [USA: Illinois]. Andersen et al. (2000: 589) [Mexico City]; Ashe and O’Connor (2009: 195). NE. Canada, Mexico, USA.

**Genus *Procladius* Skuse, 1889**

The second most speciose genus of Tanypodinae, with ~ 70 named species that occur in all zoogeographical regions except Antarctica. Four subgenera are recognized, namely *Procladius* s. str., *Holotanypus* Roback, 1982, *Laurotanypus* Oliveira, Messias & Silva-Vasconcellos, 1992, and *Psilotanypus* Kieffer, 1906 (see Ashe and O’Connor 2009; Dantas and Hamada 2018). The larvae prefer muddy substrate of standing or slow-flowing water bodies, especially ponds and small lakes, but a few also inhabit the profundal zone of large, deep lakes (Cranston and Epler 2013).

**Procladius (Psilotanypus) bellus (Loew, 1866: 4)** (*Tanypus*) [USA: Washington]. Andersen et al. (2000: 589) [Mexico City]; Ashe and O’Connor (2009: 210); Bentley and Thomas (2022) [Puebla State]. NE, OR. Canada, China, Mexico, USA.

**Procladius (Holotanypus) culiciformis (Linnaeus, 1767: 978)** (*Tipula*) [Sweden]. Andersen et al. (2000: 589) [Mexico City]; Ashe and O’Connor (2009: 199). NE, PA. Widespread.

**Remarks.** The species was recorded from Campeche State by Mendoza-Arroyo and López-Toledo (2017: 27). This record is doubtful as the specimen was studied using a stereomicroscope with too low magnification to observe morphological details and no experts were involved in the identification of the specimen.

**Genus *Psectrotanypus* Kieffer, 1909**

A genus with seven named species that occur in the Nearctic, Palaearctic, Afrotropical, and Oriental regions. The genus was recorded from the Neotropical region by Fittkau and Reiss (1979), but without specifying a country. The larvae occur in ponds, bogs, small bodies of water and slow-flowing streams (Cranston and Epler 2013).

***Psectrotanypus* sp.**: Alcocer et al. (2016: 411) [Puebla State].

**Genus *Tanypus* Meigen, 1803**

A genus of > 30 named species that occur in all zoogeographic regions except Antarctica and Oceania. Two subgenera are recognized, namely *Tanypus* s. str. and *Apelopia* Roback, 1971 (see Ashe and O’Connor 2009). The larvae live in sediments in standing and slowly flowing waters, especially in temperate to warm regions, where they can tolerate high salinity (Cranston and Epler 2013).

**Tanypus (Tanypus) catemaco (Roback, 1964: 141)** (*Pelopia*) [Mexico: Veracruz State]. Andersen et al. (2000: 589); Ashe and O’Connor (2009: 227). Endemic.

**Tanypus (Apelopia) neopunctipennis Sublette, 1964b: 118** [USA: Illinois]. Andersen et al. (2000: 589) [States of Oaxaca; Veracruz]; Ashe and O’Connor (2009: 226). NT, NE. Bahamas, Cuba (Bello-González and Téllez-Martínez 2012), Mexico, USA.

**Genus *Thienemannimyia* Fittkau, 1957**

A genus of ~ 20 named species occurring in the Nearctic, Palaearctic, Afrotropical, and Oriental regions. Unnamed species were reported from Costa Rica by Watson and Heyn (1993). The larvae are found in both lotic and lentic waters (Cranston and Epler 2013).

***Thienemannimyia* sp.**: Andersen et al. (2000: 591) [Nuevo León State].

**Genus *Zavrelimyia* Fittkau, 1962**

Syn.: *Paramerina* Fittkau, 1962.

Recently Silva and Ekrem (2016) formally placed the genus *Paramerina* Fittkau as a synonym of *Zavrelimyia* Fittkau. The genus now comprises ~ 50 named species that occur in all zoogeographic regions except Antarctica. Larvae of *Zavrelimyia* s. str. are, with few exceptions, more or less cold stenothermic and in temperate regions of the Holarctic primarily inhabitants of sandy or detritus rich sediments of springs and lentic habitats of stream sections close to springs. Larvae of Zavrelimyia (Paramerina) are eurythermic, living in a variety of standing waters of all sizes, but are also present in small lotic habitats including pools in rivers (Cranston and Epler 2013).

**Zavrelimyia (Paramerina) smithae (Sublette, 1964b: 100)** (Pentaneura (Pentaneura)) [USA: California]. Andersen et al. (2000: 589) [States of Oaxaca; Puebla]; Ashe and O’Connor (2009: 192). NE. Mexico, USA.

### ﻿Generically unplaced valid Macropelopiini

***roblesi* Vargas, 1946: 80** (*Macropelopia*) [Mexico: Chiapas State, Mariscal]. Andersen et al. (2000: 589 as *Macropelopiaroblesi* Vargas); Ashe and O’Connor (2009: 250, 362). Endemic.

### ﻿Generically unplaced valid Tanypodinae

***marmorata* Johannsen, 1938: 219** (*Pentaneura*) [Puerto Rico]. Andersen et al. (2000: 589 as *Pentaneuramarmorata* Johannsen) [States of Chiapas; Guerrero; Veracruz]; Ashe and O’Connor (2009: 252). NT. Mexico, Puerto Rico.

#### Subfamily Telmatogetoninae

**Genus *Telmatogeton* Schiner, 1867**

A genus of ~ 30 named species that occur in all zoogeographic regions. Except for a few freshwater species from Hawaii, *Telmatogeton* larvae are marine and live in the intertidal zone where they construct tubes within green algae such as *Enteromorpha* (Cranston and Ashe 2013).

***Telmatogetonalaskensis* Coquillett, 1900: 395** [USA: Alaska]. Andersen et al. (2000: 589) [Mexico, without specific locality]; Ashe and O’Connor (2009: 332). NE. Canada, Mexico, USA.

***Telmatogetonlatipenne* Wirth, 1949: 172** [Mexico: Colima State, Revillagigedo Islands]. Andersen et al. (2000: 589); Ashe and O’Connor (2009: 333). Endemic.

**Genus *Thalassomya* Schiner, 1856**

A genus of 12 named species that occur in all zoogeographic regions except Antarctica. The larvae live in the intertidal marine zone, particularly in the warmer seas of the world (Cranston and Ashe 2013).

***Thalassomyabureni* Wirth, 1949: 167** [USA: Florida]. Andersen et al. (2000: 589) [Baja California Sur State]; Ashe and O’Connor (2009: 336). NT, NE. Mexico, USA. According to Wirth (1969) distributed “from Florida to Panama and the West Indies”.

***Thalassomyalongipes* (Johnson, 1924: 86)** (*Galapagomyia*) [Ecuador: Galapagos Islands]. Andersen et al. (2000: 589) [Nayarit State: Tres Marias Islands]; Ashe and O’Connor (2009: 337). NT. Ecuador, Mexico.

***Thalassomyapilipes* Edwards, 1928: 60** [American Samoa]. Andersen et al. (2000: 589) [Baja California State; Colima State: Revillagigedo Islands]; Ashe and O’Connor (2009: 338). NT, NE, OR, AU, OC. Widespread.

### ﻿Species richness and taxonomic composition

A total of 110 species are listed for Mexico; 52 species in 25 genera belong to the subfamily Chironominae, 30 species in 13 genera to Orthocladiinae, 19 species in nine genera and two valid species that are not placed in a genus to Tanypodinae, five species in two genera to Telmatogetoninae, and two species in one genus to Diamesinae. In addition, there are records of 41 genera without identified species. Of these, 20 genera belong to Chironominae, 12 to Orthocladiinae, eight to Tanypodinae, and one genus to Diamesinae.

## ﻿Distribution

**Figure 1.**
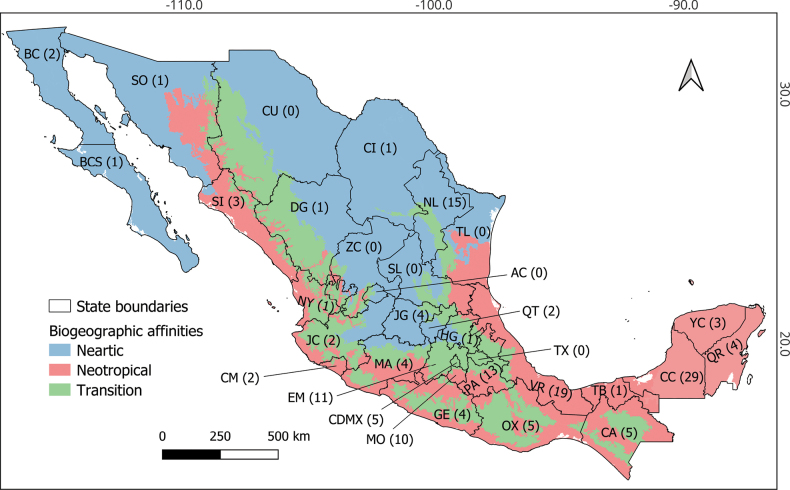
Biogeographic affinities and number of chironomid species recorded from each of the 32 Mexican states. Abbreviations: AC: Aguascalientes; BC: Baja California; BCS: Baja California Sur; CA: Chiapas; CC: Campeche; CDMX: Ciudad de México; CI: Coahuila; CM: Colima; CU: Chihuahua; DG: Durango; EM: Estado de México; GE: Guerrero; GJ: Guanajuato; HG: Hidalgo; JC: Jalisco; MA: Michoacán; MO: Morelos; NL: Nuevo León; NY: Nayarit; OX: Oaxaca; PA: Puebla; QR: Quintana Roo; QT: Querétaro; SI: Sinaloa; SL: San Luis Potosí; SO: Sonora; TB: Tabasco; TL: Tamaulipas; TX: Tlaxcala; VR: Veracruz; YC: Yucatán; ZC: Zacatecas.

The number of species recorded from the different states throughout Mexico is very uneven. More than ten species have only been recorded from six states. From Campeche a total of 29 species are recorded, most of them based on material collected during a project in Calakmul Biosphere Reserve (Contreras Ramos et al. 2000). From Veracruz 19 species have been recorded, from Nuevo León 15 species, from Puebla 13 species, from the State of Mexico 11 species and from Morelos ten species. From the remaining states only five or less species have been recorded. In most of the states in central and northern Mexico, as well as those on the Pacific coast, there are no or only a few records (Fig. 1).

The type localities for 34 Chironomidae species are in Mexico; of these, 27 species (25% of the total number of recorded species) are endemic. Twenty-nine species have a Neotropical distribution, 15 are Nearctic or Holarctic, while the remaining 39 species are distributed in both the Neotropical and Nearctic regions or are more widely distributed.

## ﻿Discussion

In addition to being key to freshwater and riparian ecosystems (e.g., Porinchu and MacDonald 2003; Paetzold et al. 2005), chironomids have been widely used to recreate the environmental history of lakes and rivers (e.g., Plikk et al. 2019), generate typologies (e.g., Schöll and Haybach 2004; Nyman and Korhola 2005), propose biogeographical hypotheses (e.g., Brundin 1966; Krosch et al. 2011), ecotoxicological models (e.g., Beleza et al. 2019; Ferrari et al. 2019), biomonitoring (e.g., Gomes et al. 2018; Molineri et al. 2020) and for the evaluation of taxonomic and functional diversity (e.g., Jyväsjärvi et al. 2018). However, the Mexican Chironomidae fauna needs to be much better studied before it can be useful in such contexts.

In the previous checklist (Andersen et al. 2000), the number of species listed was 61; so, 49 species have been added during the last two decades. Of these, no less than 25 species belong to the subfamily Orthocladiinae, and the number of Orthocladiinae species has thus increased five times from the five species recorded in 2000. In Chironominae the number of species has increased from 29 species in 2000 to 52 species today; while in the subfamilies Tanypodinae, Telmatogetoninae, and Diamesinae no species have been added since 2000.

Comparing the number of Chironomidae species recorded in Mexico with the number in other neighboring, better studied areas, highlights the need for further studies in Mexico. Oliver et al. (1990) and Oliver and Dillon (1994) listed 206 generic and 1065 species names of Nearctic Chironomidae. More than 700 species of chironomids are listed from southeastern USA, including Alabama, Florida, Georgia, North and South Carolina, and Tennessee, which together comprise approximately 41% of the total area of Mexico (Caldwell et al. 1997). For the state of California, bordering Mexico and comprising ~ 22% of the area of Mexico, 245 species of chironomids have been recorded (Spies 1999). More than 400 species have been recorded from the state of Florida, which comprises less than 9% of the total area of Mexico (Epler 2019).

No comprehensive checklist for the Neotropical region has been published since Spies and Reiss (1996). However, in an updated checklist for Brazil (Pinho 2022) 658 species in 99 genera are listed. Mendes and Pinho (2016) recently published a checklist for Colombia listing only 30 species of Chironomidae in 16 genera in three subfamilies. In addition, 32 genera and two subfamilies have been recorded from Colombia based on larva, but without identified species.

The 110 species recovered in the present checklist is far from the 1000 species estimated by Andersen et al. (2000) to occur in Mexico and highlights the need for further studies. Most additional species to be found will undoubtedly belong to the subfamilies Chironominae, Orthocladiinae, and Tanypodinae. Chironominae is the most species rich subfamily of Chironomidae and is found in all biogeographical regions except Antarctica. Additional species will mainly be found in slow flowing streams and rivers, lakes and ponds in lowland habitats, but additional species will also be found in streams, rivers and lakes at higher altitudes. Mexican species of some genera, like e.g. *Rheotanytarsus* Thienemann & Bause, 1913 have been reviewed and new species described. However, there are several species-rich genera in which Mexican material has not or hardly been studied and in genera like *Pseudochironomus* Malloch, 1915, *Tanytarsus* Wulp, 1874, and *Polypedilum* Kieffer, 1912, many more species are likely to be added. The Orthocladiinae is also a very species-rich and widely distributed subfamily that tends to be particularly abundant in streams and rivers in mountainous areas. For some genera, like *Antillocladius* Sæther, 1981, *Bryophaenocladius* Thienemann, 1934 and *Mesosmittia* Brundin, 1956, Mexican material has been included in reviews of the genera, while other species-rich genera like *Corynoneura* Winnertz, 1846 and *Cricotopus* Wulp, 1874, are hardly studied at all. Most additional Tanypodinae species will probably be found in slow flowing streams and rivers, lakes and ponds in lowland habitats. So far only a few genera of Tanypodinae have been studied in detail in Mexico and for several species-rich genera like *Ablabesmyia* Johannsen, 1905 and *Labrundinia* Fittkau, 1962, there are only a few species recorded from Mexico so far.

Particularly in Orthocladiinae, several recently described genera like *Colosmittia* Andersen & Sæther, 1994, *Litocladius* Mendes, Andersen & Sæther, 2004, and *Titimbera* Andersen, Pinho & Mendes, 2015 might also occur in Mexico as they have all been taken in Costa Rica (Andersen et al. 2011a; Mendes et al. 2011; Andersen et al. 2015). There might well be several undescribed genera in the subfamily. Epler (2017) recently recorded no less than 16 undescribed genera of Orthocladiinae from Zurquí in Costa Rica.

Additional species will also likely be found in some of the less species-rich subfamilies. Today, ten extant subfamilies of Chironomidae are recognized. Six subfamilies occur in the Nearctic region, while in the Neotropical region no fewer than nine subfamilies have been encountered. At the subfamily level the Neotropical region is thus the most diverse biogeographical region. Only the monotypic subfamily Usambaromyiinae Andersen & Sæther has not been recorded. In the Neotropical region two of the other subfamilies, Chilonomyiinae Brundin and Aphroteninae Brundin, have only been found in southern Chile and Patagonia and it is unlikely that any species in these two subfamilies occur in Mexico.

However, two subfamilies so far not recorded from Mexico might occur in the country. The subfamily Buchonomyiinae Brundin & Sæther with three included species is found in the Neotropical, Palaearctic, and Oriental regions. It was recorded for the first time from the Neotropical region by Andersen and Sæther (1995) describing *Buchonomyiabrundini* Andersen & Sæther, 1995 from a small, shallow, rather fast-flowing river in Costa Rica. The subfamily Podonominae Thienemann & Edwards has a mainly bipolar distribution with five genera and 15 species in North America and Canada, and five genera with altogether 85 species in the southern part of South America. Spies (1999) recorded two species of *Boreochlus* Edwards, 1938 and one species of *Parochlus* Enderlein, 1912 from California. Several species have recently also been described from Brazil, and two genera, *Podonomus* Philippi, 1866 and *Parochlus*, are listed from Colombia based on larvae (Mendes and Pinho 2016; Pinho 2022).

The subfamily Telmatogetoninae with two genera, *Telmatogeton* Schiner, 1867 and *Thalassomya* Schiner, 1856, is marine. Both genera with altogether five species are known from Mexico.

The subfamily Diamesinae has a mainly arctic or alpine distribution with 55 species in ten genera in the Nearctic region and 11 species in five genera in the Neotropical region. Two species of *Diamesa* Meigen, 1835 were described by Serra-Tosio (1977) from a high-altitude lake in the Mexico State. The genus, with 107 species, is known from the Nearctic, Palaearctic, Afrotropical, and Oriental regions. Spies (1999) listed six species of *Diamesa* from California and one species in each of the genera *Pseudodiamesa* Goethgebuer, 1939 and *Sympotthastia* Pagast, 1947. Based on larvae, Mendes and Pinho (2016) listed the genus *Paraheptagyia* Brundin, 1966 from Colombia. *Paraheptagyia*, with five species, is distributed in the southern part of the Neotropical region and two species occur in the Australasian region (Ashe and O´Connor 2009).

The uneven distribution of Chironomidae records throughout the states in Mexico clearly reflects the lack of Chironomidae studies. Some Nematocera groups are better studied than the Chironomidae in Mexico. Consideration of the general distribution patterns of these groups may suggest what can be expected for the chironomids. States like Oaxaca and Chiapas are among the richest when it comes to Culicidae, Simuliidae and Ceratopogonidae (Ibánez-Bernal and Coscarón 1996; Ibánez-Bernal et al. 1996). Bond et al. (2014) also demonstrated that the Pacific slope has a high diversity of aquatic insects. Climatic and topographic heterogeneity in southeastern Mexico leads to high environmental heterogeneity (Rodríguez et al. 2019). The area has a complex geology resulting in barriers such as the Isthmus of Tehuantepec that is responsible for increased diversity in several insect groups (Halffter and Morrone 2017). It is expected that future studies will show that the increase in the number of Chironomidae species will be particularly striking in Oaxaca and Chiapas.

Mexico is known to have a high proportion of endemic species. In well-studied groups like amphibians, reptiles, and mammals the proportion of endemic species is 60%, 51% and 31%, respectively (Hufnagel and Mics 2021). However, the number of records of chironomids from Mexico is clearly insufficient to appreciate patterns of endemism or clear biogeographic relationships.

To increase the number of species recorded from Mexico, taxonomic studies should be given priority. Even though rearing of larvae is important to associate the immatures with adults, chironomids are generally described based on adult males. To achieve an immediate increase in species numbers, further studies should thus focus on adults rather than on larvae and pupae. Fieldwork should be focused particularly on the states in central and northern Mexico, where the chironomid fauna is poorly known. The southeastern states along the Pacific coast should also be given special attention. Different habitats such as streams, rivers, lakes, and ponds should be visited, and collections should be made at different altitudes. Several chironomid species live in special habitats, like phytotelmata, and many species particularly among the Orthocladiinae, are semiterrestrial or terrestrial.

## ﻿Additional information

### Author ORCIDs

Orestes C. Bello-González https://orcid.org/0000-0002-7697-1618

Trond Andersen https://orcid.org/0000-0003-2201-1870

Norman Mercado-Silva https://orcid.org/0000-0001-7764-8161

### Data availability

All of the data that support the findings of this study are available in the main text.
